# Therapeutic potential of a novel multifunctional iron chelator on cognitive decicits and insulin degrading enzyme expression in a rat model of sporadic Alzheimer's disease

**DOI:** 10.1186/2050-6511-13-S1-A65

**Published:** 2012-09-17

**Authors:** Ana Knezović, Marina Knapić, Jelena Osmanović-Barilar, Silvia Mandel, Moussa Youdim, Peter Riederer, Melita Šalković-Petrišić

**Affiliations:** 1 Department of Pharmacology and Croatian Institute for Brain Research, University of Zagreb Medical School, 10000 Zagreb, Croatia; 2 Eve Topf and NPF (US) Centers of Excellence for Neurodegenerative Diseases Research, Faculty of Medicine, Technion, 31096 Haifa, Israel; 3 Department of Clinical Neurochemistry, Clinic for Psychiatry and Psychotherapy, University of Würzburg, 97080 Würzburg, Germany

## Background

There is a need in modern pharmacology for a representative animal model which should accurately mimic sporadic Alzheimer’s disease (sAD), the prevailing type of dementia in humans, and thus could be suitable for novel drug testing. Rats treated intracerebroventricularly with the betacytotoxic agent streptozotocin (STZ-icv), have been proposed recently as a non-transgenic sAD model which demonstrates AD-like pathology features at cognitive, neurochemical and structural level. In addition to the cognitive deficits, pathological accumulation of amyloid β (Aβ) peptide is one of the neuropathological hallmarks of sAD, and a growing body of evidence suggests the involvement of insulin degrading enzyme (IDE), responsible for Aβ degradation, in sAD pathophysiology. We have explored the time course of cognitive deficits and hippocampal (HPC) IDE expression in the STZ-icv rat model of sAD, and the therapeutic potential of the novel multifunctional iron-chelating drug M30 to improve these deficits.

## Methods

Adult Male Wistar rats were injected bilaterally icv with STZ (0.3, 1 and 3 mg/kg) or vehicle and sacrificed one week, or one, three, six and nine months after the treatment. Two groups of STZ-icv (3 mg/kg)-injected rats were additionally subjected to an 11-week oral M30 treatment (2 and 10 mg/kg, 3x per week) beginning 10 days after the STZ-icv treatment. Cognitive deficits were measured by the Morris water maze swimming test (MWM) and the passive avoidance test (PA). IDE protein expression in HPC was measured by SDS-PAGE electrophoresis/immunoblotting. Data were analysed by the Kruskal-Wallis and the Mann-Whitney U test (p < 0.05).

## Results

STZ-icv rats exhibited significant dose- and time-dependent cognitive deficits in the PA test (40–90%), while IDE protein expression was found to be decreased not earlier than one month after the STZ-icv administration (−56%), persisting decreased untill six months (−26%). Treatment with the high M30 dose improved STZ-icv-induced cognitive deficits, observed as a decreased number of mistakes in the MWM test (−60%) and increased latency time in the PA test (+300%). Treatment with both M30 doses significantly increased IDE protein expression in comparison with the STZ-icv treatment alone (low dose +19%, high dose +37%).

## Conclusions

The STZ-icv rat model demonstrates long-term cognitive deficits and decreased hippocampal IDE protein expression which tend to correlate mutually. Chronic M30 treatment, initiated after the development of cognitive deficits, significantly improves the cognitive deficits as well as decreases IDE protein expression in the STZ-icv rat model of sAD, suggesting that multifunctional iron-chelating drugs might have a therapeutic potential in sAD treatment.

